# Shaping the future of pediatric rheumatology

**DOI:** 10.1186/s12969-024-00957-5

**Published:** 2024-01-16

**Authors:** Tadej Avčin, Angelo Ravelli

**Affiliations:** 1 Department of Allergology, Rheumatology and Clinical Immunology, University Children’s Hospital, University Medical Center Ljubljana, Bohoričeva 20, SI-1525 Ljubljana, Slovenia; 2 Department of Pediatrics, Faculty of Medicine, University of Ljubljana, Ljubljana, Slovenia; 3 IRCCS Istituto Giannina Gaslini, Genoa, Italy; 4 Department of Neurosciences, Rehabilitation, Ophthalmology, Genetics and Maternal-Infantile Sciences, University of Genoa, Genoa, Italy

Dear colleagues,

It is with great pleasure and honor that we address you as the newly appointed Editors-in-Chief of *Pediatric Rheumatology*, an open access journal published by BioMed Central, a member of the Springer Nature publishing group. The journal has been serving the worldwide pediatric rheumatology community for more than two decades and was originally launched as the *Pediatric Rheumatology Online Journal* in 2003 as the first solely pediatric rheumatology journal. In 2007, the journal was renamed *Pediatric Rheumatology* [1] and received its first impact factor in 2012 [2]. Throughout all these years the journal remained freely accessible and served the pediatric rheumatology community to share and exchange advances in clinical care, research and education.

The great progress of the journal since its foundation is a legacy of the remarkable past Editors-in-Chief, Prof. Charles H. Spencer and Prof. Alberto Martini, who established and developed the journal, which is now read by pediatricians, pediatric rheumatologists, adult rheumatologists, healthcare professionals, and the general public. We would like to express our heartfelt thanks to the past Editors-in-Chief for their vision, dedication, and excellence in the continuous improvement of the journal. We would also like to thank all the authors, readers, reviewers, former members of the editorial board and other supporters who have contributed to the significant success and growth of the journal in the past years.

*Pediatric Rheumatology* has been an official journal of the Paediatric Rheumatology European Association (PReS) since 2013 [3]. With the support of PReS, the journal had a direct link with practicing pediatric rheumatologists and researchers, which proved to be a fruitful partnership for both parties. PReS provided also financial support to the journal to maintain a competitive price for authors with limited funds to publish their work. In the last decade, PReS has evolved into a truly global society, which was also reflected by the journal. PReS has established close links with other societies and networks, such as PRINTO, CARRA, ERN-RITA, EULAR, and others, to form a worldwide network of professionals working in the field of pediatric rheumatology. We believe that ongoing close partnership between *Pediatric Rheumatology* and PReS is instrumental to cover cutting–edge advances in research and clinical care of children with rheumatic diseases, and providing a platform for international cooperation.

We accepted the roles of the new Editors-in-chief with a vision to foster further growth of the journal and to provide a tool for the exchange of information within a broad, vibrant, and inclusive pediatric rheumatology community. Our goal is to provide global solutions for the management of pediatric rheumatic diseases, promote innovative research, advance education, and raise awareness of pediatric rheumatic diseases. To ensure a stable progress of the journal and provide the highest quality standards, we appointed a new editorial team with a wide international representation of esteemed pediatric rheumatology practitioners and researchers who will oversee the journal’s content and peer-review process. In addition, the Editorial board has been expanded to include four Section Editors (Michael W. Beresford, Christoph Kessel, Raju Khubchandani, and Susan Shenoi) that will have a decisive roles in shaping the journal and will cover specific fields including clinical and translational research, global clinical practice and educational aspects.

*Pediatric Rheumatology* will continue to cover the full spectrum of pediatric rheumatology and will maintain its strengths with a patient-centered approach. The journal will remain open to both clinical and translational research, illustrative case reports, reviews, and commentaries. Moreover, in line with our vision, we would like to introduce some new initiatives that aim to enhance the journal’s impact factor through the involvement and help of the entire international pediatric rheumatology community. To address this need, we will support the following:

- Increasing diversity of publications, reviewers and editors to ensure the highest quality of the journal.
- Publication of clinical practice guidelines and recommendations developed through the collaborative efforts of PReS, CARRA, and other networks.
- Promote translational research in pediatric rheumatology.
- Introduce a dedicated section that focuses on clinical insights, best practices, and educational case studies.
- Introduce a series of review articles based on lectures at the PReS courses and presentations at the annual PReS congresses.
- Encourage participation of patients, caregivers, and policymakers to share their views, insights, and suggestions.

Above all, we would like to develop the journal according to the needs of the community and please email us any other suggestions, or feedback on how we can further improve it to serve as a valuable resource to our readers.

We are committed to making research freely available to readers worldwide in an open access format, which enables growth of pediatric rheumatology in both developed and developing countries. The model of an Open Access journal was supported by 80% of pediatric rheumatologists who replied to a survey in 2002 [3]. Of course, open access publishing comes with article processing charges (APC), which we would like to keep in a competitive range. The Publisher also has membership arrangements with many institutions to enable authors to publish for free or at a discounted rate. In addition, the publisher provides support for corresponding authors based in the world’s low-income countries and lower-middle-income countries. Requests for APC waivers and discounts from other authors will be considered on a case-by-case basis and may be granted in cases of financial need as well. Moreover, we would like to enable full or partial discounts of publication fees for the collaborative work of PReS working parties and others, such as publications of new recommendations on the management of pediatric rheumatic diseases and papers from funded projects (i.e. PReS-CARRA and PReS-PRINTO grants) that have limited resources. Please check the information on the funding support services at the journal’s website and how to get in touch if you have any questions.

We look forward to shaping our journal together in the future and encourage you to submit your work, share your expertise, engage in discussions, and spread the word.

Thank you for your ongoing support.
